# Evaluation of EMG processing techniques using Information Theory

**DOI:** 10.1186/1475-925X-9-72

**Published:** 2010-11-12

**Authors:** Fernando D Farfán, Julio C Politti, Carmelo J Felice

**Affiliations:** 1 Laboratorio de Medios e Interfases, Departamento de Bioingeniería, Universidad Nacional de Tucumán, Consejo Nacional de Investigaciones Científicas y Técnicas. San Miguel de Tucumán, Argentina

## Abstract

**Background:**

Electromyographic signals can be used in biomedical engineering and/or rehabilitation field, as potential sources of control for prosthetics and orthotics. In such applications, digital processing techniques are necessary to follow efficient and effectively the changes in the physiological characteristics produced by a muscular contraction. In this paper, two methods based on information theory are proposed to evaluate the processing techniques.

**Methods:**

These methods determine the amount of information that a processing technique is able to extract from EMG signals. The processing techniques evaluated with these methods were: absolute mean value (AMV), RMS values, variance values (VAR) and difference absolute mean value (DAMV). EMG signals from the middle deltoid during abduction and adduction movement of the arm in the scapular plane was registered, for static and dynamic contractions. The optimal window length (segmentation), abduction and adduction movements and inter-electrode distance were also analyzed.

**Results:**

Using the optimal segmentation (200 ms and 300 ms in static and dynamic contractions, respectively) the best processing techniques were: RMS, AMV and VAR in static contractions, and only the RMS in dynamic contractions. Using the RMS of EMG signal, variations in the amount of information between the abduction and adduction movements were observed.

**Conclusions:**

Although the evaluation methods proposed here were applied to standard processing techniques, these methods can also be considered as alternatives tools to evaluate new processing techniques in different areas of electrophysiology.

## Background

The electromyographic (EMG) signal measures electrical currents generated in muscles during its contraction representing neuromuscular activities. It is a result of the summation of all Motor Unit Action Potentials (MUAP) in the region near the electrodes. The composition of a surface EMG signal from MUAPs results in a stochastic signal because of the different firing rates and the large number of motor units that contribute. Raw EMG offers valuable information in a particularly useless form. This information is useful only if it can be quantified.

Analysis of EMG signals with powerful and advanced methodologies is becoming a very important requirement in biomedical engineering. The main reason for the interest in EMG signal analysis is in clinical diagnosis and biomedical applications [1]. Recent advances in technologies of signal processing and mathematical models have made it possible to develop advanced EMG detection and analysis techniques. In the time domain, two parameters are commonly used: the root-mean-squared (RMS) value and the average rectified value. Both are appropriate and provide useful measurements of the signal amplitude [2-4]. Various mathematical and Artificial Intelligence techniques have received extensive attention [5]. Mathematical models include wavelet transform, time-frequency approaches, Fourier transform, Wigner-Ville Distribution, statistical measures, and higher-order statistics [1].

The selection of an appropriate processing technique depends on the physiological characteristics of the muscles that are desired to study. That is, processing techniques that provide information about the content in frequency of the EMG must be used, if it is wanted to analyze the muscle fatigue, (e.g. power spectral density, PSD). If it is wanted to analyze the recruitment of muscle fibers during a contraction, estimators of EMG amplitude would be more appropriate (e.g. RMS). However, for election of an appropriate processing technique these criteria are not sufficient, since an estimate of EMG amplitude can be done with many processing techniques.

In this paper two methods for evaluation of processing techniques based on information theory are proposed. One of them is designed to evaluate techniques in static contractions and the other one in dynamic contractions. Both methods of evaluation allow to measure the "mutual information" [6,7], hereafter referred to as "information." Using this framework, the maximum amount of knowledge (the upper bound of information) available to an observer who "reads off" the EMG signals can be determined. These methods were inspired by the works of Arabzadeh et al (2006) and Rogers et al (2001) [8,9].

The EMG of muscle middle deltoid is used because it is the main protagonist in the shoulder abduction and adduction movements. Four well-known techniques are evaluated to determine which one is the most adequate to monitor and quantify variations in the EMG signal amplitudes. These techniques were: RMS value, Variance value (VAR), absolute mean value (AMV), difference absolute mean value (DAMV) [10,11].

Generally, a segmentation of EMG signal is required when some processing technique is implemented. In myoelectric control a segment is a time slot for acquiring myoelectric data considered for feature extraction. Due to real-time constraints, an adjacent segment length plus the processing time of generating classified control commands should be equal or less than 300 ms [12]. Furthermore, a segment length should be adequately large, since the bias and variance of features rise as segment length decreases, and consequently degrade classification performance. The optimal window length had been determined (optimal segmentation) for static and dynamic contractions, using the proposed evaluation methods.

The information values were also used to analyze the abduction and adduction movements and the influence of inter-electrode distance on dynamic contractions.

Here, the evaluation methods were applied to standard processing techniques, however, these can also be considered as alternatives tools for evaluation of new processing techniques in different areas of electrophysiology (e.g. electromyography, electroencephalography, electroneurophysiology, and others) as well as to quantify the hysteresis effect given in muscular systems [13] and their dependence on different experimental situations such as movements under external loads, speed of movement and muscle fatigue. Moreover, it would also determine the degree of correlation between the stimulus and response in different phases of movement. This latter study would be of particular interest in applications such as myoelectric control.

## Methods

### Electromyogram recordings

**Figure 1:**
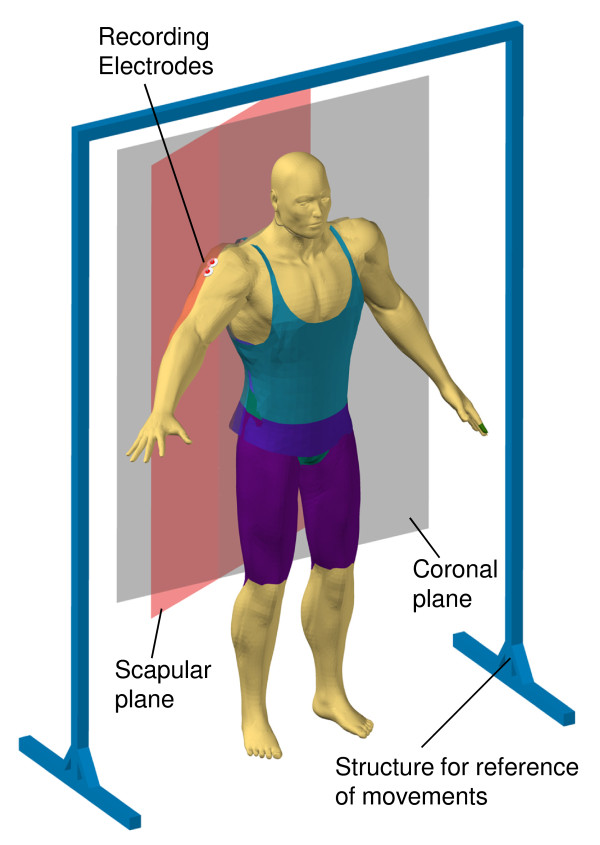
**Movement protocols, and EMG recordings during static and dynamic contractions of middle deltoid. **Static contractions. The positioning of the arm was done through abductions movements in the scapular plane. This plane is 30° of coronal plane. The structure for reference of movements allows the correct global positioning (posture) among experimental subjects. A custom made angular positioning device was attached to the frame of reference. The movable part of angular positioning device is displaced by the arm of subject. The fixed part has a gradual scale with the positions of 0° to 90° in step of 10°. The recording electrodes are placed in the middle deltoid (bipolar configuration). The reference electrode is placed in the forearm of the subject. Dynamic contractions. Dynamic contractions were evoked by abduction and adduction movements in the scapular plane. A custom made angular displacement sensor was attached to each experimental subject for monitoring of arm position.

The EMG signals from the middle deltoid were registered during static and dynamic contractions. These contractions were produced by arm abduction and adduction movements in the scapular plane (Figure 1). Each subject stood up with his/her shoulder rotated 20° forward with respect to the saggital plane, to make the movements on the scapular plane and so decreasing muscular work, basically for the middle deltoid muscle activity during abduction and adduction [13-15]. This muscle was preferred because, in the scapular plane, its action can be taken as similar to a string running on a pulley when raising or lowering a weight, say, the arm during the abduction-adduction movements. Besides, this muscle is easily accessible to placing the electrodes. We followed the SENIAM recommendations to ensure reproducible electrode placement on middle deltoid. These were placed between the acromion and the lateral epicondyle of the elbow (the greatest bulge of the muscle).

The EMG signal was amplified using a BIOPAC EMG100B electromyogram amplifier module with a high pass filter (10 Hz) and a low-pass filter (500 Hz). Arm angular displacements in the scapular plane were acquired with a BIOPAC AD100 universal amplifier module. The EMG signals and the angular displacements (dynamic contractions) were acquired at a rate of 2 kHz by a BIOPAC MP100WSW system with a 16 bits A/D resolution. The configuration of the acquisition parameters (sample rate, channels configuration and recording time) was performed with AcqKnowledge software for windows.

The EMG signals were recorded using two superficial electrodes, placed on the right middle deltoid, separated 2 cm between them. The reference electrode was placed on the forearm.

#### Static Contractions

Ten healthy subjects (7 male and 3 females) ranging in age from 23 to 48 years (mean = 35 years; standard deviation [SD] = 9.8 years) participated in the study after giving written informed consent. Ten contraction levels of middle deltoid were induced through pre-established positions of the arm. These positions were from 0° to 90°; 10° by step, and it were carried out through abduction and adduction movements depending on the initial position of the arm. That is, the positions of 0° to 90°, 10° by step, will be established by abduction movements, whereas the positions of 90° to 0°; 10° by step by adduction movements. EMG records were obtained at each angular static position during three seconds with intervals of six seconds to permit the subject the positioning of the arm at the new angle. Here we have considered both positioning movements because the EMG amplitude of medium deltoid, during a static contraction, varies according to movement used to position the arm [13].

#### Dynamics Contractions

EMG signals were registered from the middle deltoid, in ten normal subjects (6 male and 4 female, whose ages were from 20 to 31 years) during dynamic contractions induced by arm abduction and adduction movements. The arm was moved voluntarily from the rest position (0°) with an abduction movement to the maximum muscular contraction position (90°) at a constant speed (about 4.5°/sec). For this, a visual feedback was implemented. The total duration of each movement was between 45 sec and 50 sec. Each subject performed between 5 and 10 training movements (using the visual feedback) before EMG recordings were recorded for further processing. The position of the arm was continuously monitored with an angular displacement sensor based on a linear potentiometer. The abduction and adduction movements were repeated five times for each subject.

#### Variation of the inter-electrode distance

The variation of the inter-electrode distance was studied in dynamic contractions. In this case the arm was moved voluntarily from the rest position (0°) with an abduction movement to the maximum muscular contraction position (90°). The EMG signal was acquired using bipolar electrodes whose inter-electrode distances were 2, 4 and 6 cm. The abduction and adduction movements were repeated five times for each inter-electrode distances. The EMG signals obtained during dynamic contractions were recorded simultaneously for all inter-electrodes distances. Therefore, the movement speed during "variation of inter-electrode distance" was the same as "dynamic contractions". The electrodes were placed so that the center remains at the same place for all three IEDs.

### Digital processing

#### Information theoretic analysis of EMG

The proposed method for calculation of the information requires a pair of stimulus/response situations (minimum condition). Usually, the stimulus can be a time series (arm angular displacement) or simply belong to a class (e.g. position1, position2 position3, ...). The response depends of the time series characteristics that are analyzed (amplitude, frequency, number of MUAPS, ...). Thus, the responses may be: real values (RMS values, mean frequency, ...) or whole values (number of MUAPs).

Next two proposals are detailed to determine the amount of information: the first proposal is applied to static contractions while the second is applied during dynamic contractions.

#### Information in static contractions

The information that has the EMG (response) about the contraction level (stimulus) can be quantified by the information equation of Shannon [6]. In abbreviated form, in:

(1) $$I=\sum_{}P(r).P(s|r)\log_{2}\frac{P(s|r)}{P(s)}$$

Where *P(s) *is the probability of presentation of stimulus *s*, *P(s|r) *is the conditional probability of *s *given observation of response *r*, and *P(r) *is the probability of response *r *unconditional on the stimulus.

From eq. 1 it is observed that the main problem to obtain the amount of information is to determine all probability distributions. The stimulus are classified according to the arm position: *pos_0°_*, *pos_10°_*; *pos_20°_*,..., *pos_90°_*. The responses of middle deltoid muscle are given by real numbers (e.g. RMS value).

**Figure 2:**
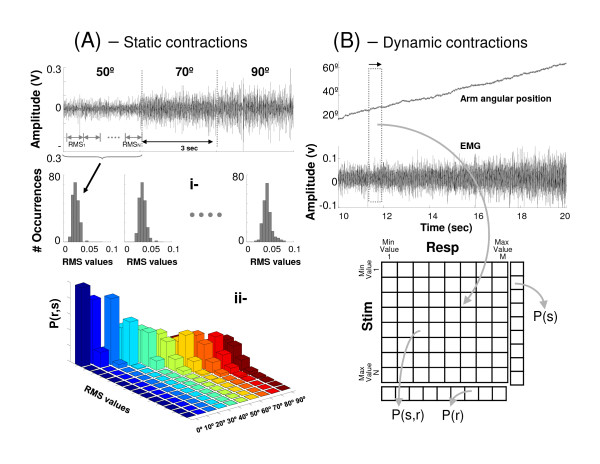
**Schematic representation of analytical method. **(A) The amplitude values of EMG signal (e.g. RMS) were determined using a segmentation. Then, from frequency diagrams of each experimental situation (i-) the joint probability distribution (ii-) was determined. (B) A window (shaded vertical rectangle) of fixed width is slid over the EMG of middle deltoid and arm angular position (top trace). At each window position, the amplitude value of EMG signal and the average stimulus value are used to increment the appropriate entry in a co-occurrence matrix, which defines the joint probability distribution, P(s,r). The stimulus and response probability distributions (P(s) and P(r), respectively) are given by the marginal distributions of the co-occurrence matrix.

In summary, the procedure is as follows:

• *Determination of frequency diagrams*. For each experimental situation (arm position) the histogram of the response variable is determined (Figure 2A).

• *Determination of joint probability distribution P(s,r)*. An [N × M] array is formed. Where N are the possible responses of the system (e.g. values range that can take the EMG amplitude) and M are the stimuli applied to the system (angular positions related to contraction levels). Thus, each element of the [N × M] array is the joint probability value *P(s,r)*.

• *Determination of probabilities distribution P(r) and P(s)*. The probability of response *r *unconditional on the stimulus *s*, is called the marginal probability *P(r) *and it can be calculated by performing the sum of joint probabilities for a given response. This is:

(2) $$P(r)=\sum_{i=1}^{M}P(s_{i},r)$$

• *Determination of conditional probability distribution P(s|r)*. The conditional probability for each stimulus can be obtained from joint and marginal probability distributions. The conditional probability is given by:

(3) $$P(s|r)=\frac{P(s,r)}{P(r)}$$

Thus, *P(s|r) *is obtained for each contraction level: *P(s_0°_|r), P(s_10°_|r),..., P(s_90°_|r)*.

• *Determination of information*. Finally, the information is calculated using eq. 1.

#### Information in dynamic contractions

The arm angular position (stimulus) and EMG signal were discretized into time segments of equal length without overlapping. At each successive window position, one specific attribute of both the stimulus (its mean value) and the EMG signal (for example, the RMS value in the window) was measured and used to increment the appropriate bin in a co-occurrence matrix, created as follows. First, the maximum and minimum values of these attributes were determined, and their distributions were divided into bins of equal width (Figure 2B). The stimulus-response co-occurrence matrix could contain M response columns, which could vary from M = 2 up to M = n + 1, where n equals the maximum RMS vale observed in that sliding window width at any window location. The stimulus distribution was divided equally into N rows, where N ≥ 2. The greater number of categories required to divide the dynamic range of the stimulus into bins of 5° (arm angular position) was set as an upper limit on N. Therefore the three free parameters are the number of discrete categories into which the stimulus and response are divided, as well as the window length. The window length was established in the range of 20 ms to 2000 ms for analysis of segmentation time. Then an optimum segmentation time was fixed for evaluation of processing techniques.

The co-occurrence matrix defines the joint probability distribution, *P(s,r)*. The stimulus and response probability distributions [*P(s) *and *P(r)*, respectively] are given by the marginal distributions of the co-occurrence matrix. The conditional probability distribution, *P(s|r)*, is calculated using the eq. 3. Then by using these probabilities distributions information was calculated with eq. 1.

### EMG - Digital Processing

To next, the processing techniques evaluated in this paper are described. The choice of these techniques was realized considering previous works [2,12,16,17].

• RMS value (Root Mean Square) [10].

(4) $$RMS=\sqrt{\frac{1}{N}\sum_{k=1}^{N}{\left[x_{k}\right]}^{2}\;}\text{k}=\text{1},\text{2},\;....,\text{N}\;\;\;\;\;\;\;\;\;\;\;\;$$

N: number of samples, *x_k_*: the *k*-sample.

• Absolute Mean Value (AMV).

(5) $$\bar{x_{i}}=\frac{1}{N}\sum_{k=1}^{N}\left|x_{k}\right|$$

*x_k _*: the *k*-sample in the segment *i*.

• Difference Absolute Mean Value (DAMV).

(6) $$\bar{\Delta x_{i}}=\frac{1}{N-1}\sum_{k=1}^{N-1}\left|x_{k+1}-x_{k}\right|$$

• Variance Value (VAR)

(7) $$\sigma_{i}^{2}=E\left\{x_{i}^{2}\right\}-E^{2}\left\{x_{i}\right\}$$

*E*{*x_i_*}: the expected value of the signal in the segment *i*.

### Procedures and analysis

#### Optimal segmentation

Generally, a segmentation of EMG signal is required when some processing techniques is implemented. This segmentation process consists of dividing the temporal series into intervals with or without overlapping. In this paper we have evaluated the optimal window length (optimal segmentation). The window lengths were analyzed in the range of 20 ms to 1000 ms by step 10 ms (static contractions) and 20 ms to 2000 ms by step 10 ms (dynamic contractions). For this, the EMG recordings in positions of 0 ° to 90° (reached with abduction movements) have been used. Here, the system's responses were RMS values of EMG signal. Discretization of the response (RMS values) was performed according to the criteria frequently used in the construction of histograms: the class number is approximately the square root of the number of data. The number of data depends on the segmentation used. Thus, an information value (in bits) was obtained for each segmentation.

#### Evaluation of processing techniques

Having established the optimal segmentation, the processing techniques (mentioned above) were evaluated by calculating the amount of information extracted from each of them.

#### Analysis of abduction and adduction movements

The abduction and adduction movements were analyzed using RMS value of EMG signal and an appropriate segmentation.

#### Influence of inter-electrode distance

This study was carried out by calculating the amount of information for each inter-electrode distance. For this we used the optimal segmentation and the processing technique that provides more information. The abduction and adduction movements were considered in this analysis.

The digital signal processing of the EMG was implemented in MATLab.

## Results

The abduction movement speed was obtained from the angular displacement signal and it was 4.6 degree per second. The arm remained at 90° during 4 sec. Then the arm immediately returned to rest position with an adduction movement at the estimated speed of 4.5 degree per second. The total duration of each trial was 45 sec.

### Optimal segmentation of EMG signal

**Figure 3:**
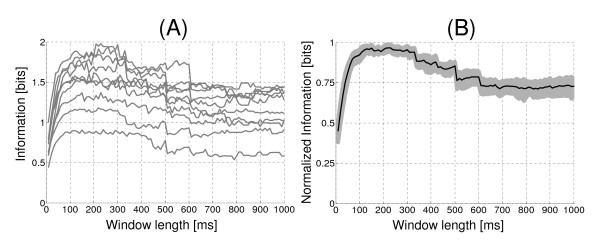
**Effect of window length (segmentation) on amount of information in static contractions. **(A) Information versus window length for each experimental subject. The RMS values of EMG signal were used for the calculation of the information. (B) Average normalized information from all subjects (continuous black line) versus window length (ms). The gray area indicates the standard deviation of the normalized information of all experimental subjects.

The optimal window length of EMG (segmentation) was computed for static and dynamic contractions. Figure 3A shows the amount of information versus window length for each of the experimental subjects. In this figure can be seen that the maximum amount of information per subject is approximately in the range of 100 ms to 300 ms. To determine the optimal window length, we normalized the information values to its corresponding maximum value (for each subject). Thus, the average ± standard deviation of all curves normalized information versus window length was obtained. Henceforth, the information values refer to average information values obtained from all subjects. It is observed in Figure 3B that the maximum information is in the range of 120 ms to 300 ms. In this interval time the information has a value of (1.50 ± 0.31) [bit].

The information values versus window length were also analyzed using other processing techniques (AMV, DAMV and VAR). The maximum amount of information was found in a smaller range of segmentation (from 100 ms to 200 ms) using AMV and DAMV. While using VAR, the optimal segmentation range is similar to the found with RMS. The information values were (in the segmentation range of 120 ms to 300 ms): (1.44 ± 0.32) [bits] using AMV, (1.50 ± 0.32) [bits] with DAMV and (1.12 ± 0.26) [bits] with VAR.

**Figure 4:**
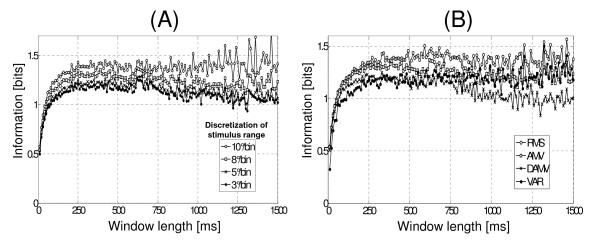
**Effect of window length on amount of information in dynamic contractions. **(A) Information vs window length. The amount of information obtained by discretizing the stimulus (arm position) was calculated using 3°/bin, 5°/bin, 8°/bin and 10°/bin. The response discretization (idem to static contractions) was as follows: the number of classes (bins) is approximately the square root of the number of data. (B) Information versus window length obtained for each processing technique. In this case, stimulus discretization was 5°/bin, while response discretization was similar to that described above.

To calculate the amount of information in dynamic contractions, stimulus and response discretization are necessary. The stimuli discretization used in this paper were 3°/bin, 5°/bin, 8°/bin and 10°/bin. The response was discretized using the square root of the number of data (this is the same criterion used in static contractions). Figure 4A shows the influence of EMG segmentation and stimulus discretization, on the amount of information. The maximum amount of information is achieved by using segmentations larger than 250 ms. It is also observed that the information increases with the stimulus discretization

Figure 4B shows the effect of the segmentation on the amount of information, using different processing techniques.

### Evaluation of processing techniques

**Figure 5:**
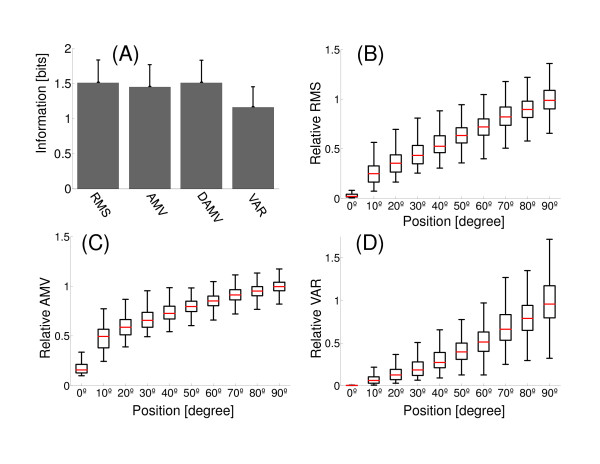
**Evaluation of processing techniques in static contractions. **(A) Amount of information for each processing technique. A segmentation of 200 ms was applied to EMG signal. (B) Box plot diagrams of the distribution of relative RMS for the static positions of the arm. The relative values were calculated according to the average maximum value. (C) Box plot diagrams of the distribution of relative AMV; (D) Box plot diagrams of the distribution of relative VAR.

To calculate the information we used a window length of 200 ms (400 samples) in static contractions and the response discretization was performed by dividing the range of it in $\sqrt{N}$bins, where N is the number of data. Figure 5A shows the amount of information evaluated for each processing technique. RMS, AMV and DAMV provided the highest values of information. In these cases the differences in the information values were not significantly different from each other (ANOVA, p = 0.89, p > 0.01). However it can be seen in Figure 5B and 5C that the RMS has a more linear behavior (with the arm position) than with the DAMV and AMV techniques (DAMV has a behavior similar to AMV). VAR provides the least amount of information. Despite having a linear behavior (in average), the dispersion of VAR values has an incremental behavior with the arm position (Figure 5D).

For the evaluation of processing techniques in dynamic contractions, the EMG signal was segmented using window length of 300 ms (600 samples) without overlapping. Stimulus discretization (arm position) was performed using 5°/bins.

**Figure 6:**
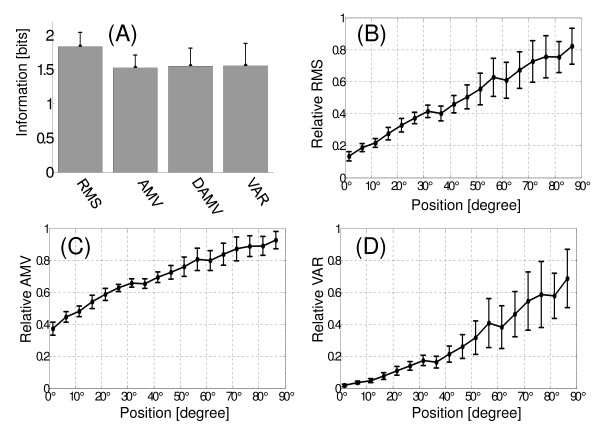
**Evaluation of processing techniques in dynamic contractions. **(A) Amount of information for each processing technique. A segmentation of 300 ms was applied to EMG signal. Stimulus discretization (arm angular position) used was 5°/bin, while response discretization was according to number of data N ($\sqrt{N}$). (B) [MEAN_RMS _± STD_RMS_] versus arm angular position. (C) Idem to B, using the average of AMV values normalized. (D) Idem to B, using the average of VAR values normalized.

The information values obtained with the different processing techniques are shown in Figure 6. As in static contractions, the RMS presents the major amount of information (1.83 ± 0.21) [bits]. This information is significantly different from AMV, DAMV and VAR techniques (ANOVA, p < 0.01). Qualitatively, can be seen that the RMS is more linear than other processing techniques. The VAR values increase exponentially (Figure 6B,6C and 6D).

### Abduction and adduction movements

**Figure 7:**
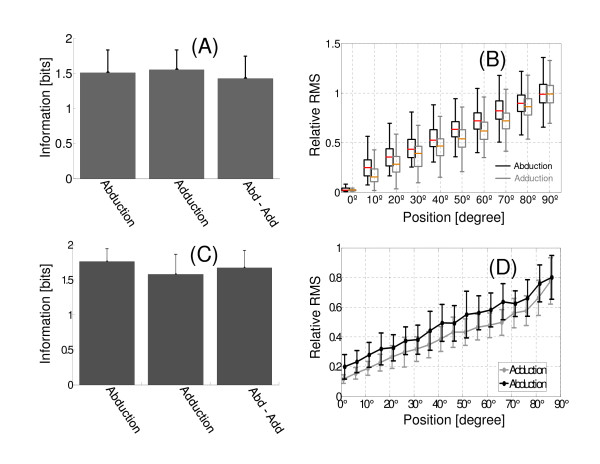
**Amount of information in abduction and adduction movements. **(A) Information obtained in static contractions. (B) Box plot diagrams of the RMS values distribution for the static positions of the arm (for all subjects). (C) Information obtained in dynamic contractions. The stimulus discretization was 5°/bin, while response discretization was according to number of data N ($\sqrt{N}$). (D) [MEAN_RMS _± STD_RMS_] versus arm angular position.

Figure 7A shows a bar graph with three averages information values with their respective variations (standard deviations). The average information values were calculated using only the arm positioning with abduction movements (label bar: Abduction), only adduction movements (label bar: Adduction) and using the positioning with both movements (label bar: Abd-Add). In the latter case, the duration of EMG recordings in each arm position would be 6 sec, the first 3 sec when the arm was positioned with an abduction movement and the other 3 sec when an adduction movement was used. The average information values did not show significant differences (ANOVA, p > 0.01).

RMS value distribution versus arm angular position is shown in Figure 7B. These values were normalized with respect to its average maximum value (for each subject). These average maximum values were obtained as follows: a) RMS values are determined (AMV, DAMV or VAR, according to the processing technique that is being evaluated) for maximum contraction. In static contractions, this situation has 3 sec duration, whereas in dynamic contractions has about 4-5 sec (arm angular position: 90°). b) Because in each case a EMG segmentation is used, N RMS values are obtained. Then these are averaged. c) Then, RMS values for the other contraction levels are normalized from the average maximum value. The medians of the RMS distributions have a linear behavior with respect to arm position. These values were higher in abduction than in adduction movements for all arm positions.

In dynamic contractions, the amount of information obtained during abduction movements does not present significant differences with adduction movements (p > 0.01) (Figure 7C). No statistically significant changes when both movements were considered in the calculation of the information. However, the average information value for Abd-Add, it decreased compared to the Abd.

### Inter-electrode distance

**Figure 8:**
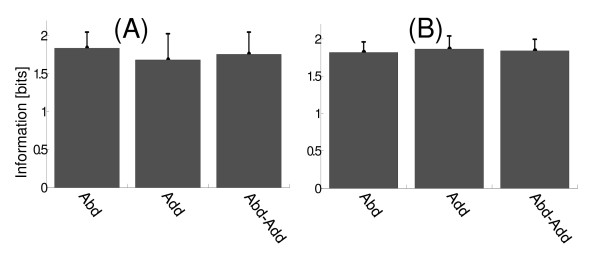
**Effect of inter-electrode distance on the amount of information. **In these cases, EMG signal was segmented using a window length of 300 ms. RMS was used. Information values were calculated by considering abduction (Abd), adduction (Add) and both movements (Abd-Add). The arm position was discretized using 5°/bins, while the response discretization was realized according to number of data N ($\sqrt{N}$). (A) Information values for IED of 4 cm. (B) Information values for IED of 6 cm.

The effect of inter-electrode distance (IED) on the amount of information was analyzed for each arm movement as well as for the combined movement (abduction and adduction movements). An increase of information with the inter-electrode distance is observed (Figure 8A). We used a window length of 300 ms for EMG signal segmentation (without overlapping). Then, RMS values were calculated in each window. By increasing the inter-electrode distance, of 2 cm to 4 cm, the amount of information increases from (1.76 ± 0.18) [bit] to (1.84 ± 0.21) [bit] for abduction movements (Figure 7C and Figure 8A). When the inter-electrode distance changes from 4 cm to 6 cm, the amount of information decreases to (1.81 ± 0.14) [bits]. This information change is not statistically significant.

In the case of contractions evoked by adduction movements it is observed that information values increase significantly in all cases, resulting in (1.58 ± 0.29) [bit], (1.67 ± 0.35) [bit] and (1.86 ± 0.18) [bits] for 2, 4 and 6 cm, respectively (Figure 8). An increase in amount of information with IED is observed when both movements (Abd-Add) are considered ((1.67 ± 0.25) [bit], (1.75 ± 0.29) [bit] and (1.84 ± 0.16) [bits] for 2, 4 and 6 cm, respectively).

## Discussion

In this paper we used information theory to design two methods for evaluation of EMG processing techniques. Because the main acting muscle during abduction and adduction movements (but admittedly not the only one) is the middle deltoid, we used its EMG signal [9]. The first proposed method allows to calculate the information value in static contractions, while the second in dynamic contractions.

Before evaluating the different processing techniques, we first obtained the optimal segmentation. This value was defined as the window length for which the maximum amount of information is obtained. Then, processing techniques were evaluated using the optimal segmentation. Finally, abduction-adduction movements and inter-electrode distance were analyzed using the best processing technique. Next, we discuss the results.

### 

#### Segmentation

The segmentation of the EMG signal is an important procedure in the processing. In this paper we showed that the optimal EMG segmentation obtained in static contractions differs of the dynamic contractions (200 ms and 300 ms, respectively). However, these results may vary according to the amount of muscles involved in movement.

Some authors have proposed segmentations of 60 ms to 500 ms. This criterion is based on which the EMG signal can be considered quasi-stationary in these windows length. However, this quasi-stationary is dependent on the signal spectrum properties [18-20]. St-Amant et al, (1998) investigated the effect of the window length on the computed RMS of EMG [21]. They assumed that the muscle activity was constant during the recording and were able to demonstrate that a longer window length can significantly increase the signal to noise ratio performance. But a very large window would average the signal over time and all variation with time information would be lost [21]. In this paper, this behavior would correspond to the range 20 ms to 200 ms, in which an increment of information is observed (related to an increase in signal to noise ratio). On the other hand our results show that an excessive increase of window length (greater than 300 ms) not causes significant increases in information (although in some cases causes a decrease of information).

The evaluation methods proposed in this paper allow obtain the optimal window length considering all the inherent characteristics of the EMG signal (such as static and dynamic contractions, kinematic characteristics of motion such as speed and acceleration, muscles involved in the movement).

#### Evaluation of processing techniques

In general processing techniques extract different features of the EMG signal. Thus, for example, the EMG amplitude can be estimated using RMS, AMV, DAMV, VAR, and others. The evaluation methods proposed in this paper provide the amount of information that a processing technique is able to extract of the EMG signal. This makes it possible to identify the characteristic of response most associated with the stimulus applied.

In this paper we found that processing techniques that provide more information were RMS, AMV and DAMV in static contractions, and only RMS in dynamic contractions. The amount of information would indicate the relation degree between stimulus and response, but does not provide information about the behavior of the response with the stimulus. This particularity can be observed in Figure 5, where processing techniques have different behavior, although the information values are significantly similar. In this figure, the medians of RMS values show a linear behavior with the arm position. It is also observed that the average variability of the RMS values is maintained constant in all positions. On the other hand, the medians of AMV values vary logarithmically with the arm position. The variability of AMV values differ with arm position. The VAR values vary exponentially and its variability presents an incremental behavior with the arm position. Thus, this latter behavior would cause a decrease in the amount of information as shown in Figure 5A.

In dynamic contractions the information increases with the degree of discretization of stimulus. This behavior is intuitively correct, since it is easier to differentiate two remote stimuli (e.g. 10°/bin) than two near stimuli (e.g. 3°/bin). The EMG signals can be used to control the movement and/or assistive devices (myoelectric control). In these cases, the on-off control implementation could require a high discretization, since it would permit better differentiation among stimuli. However, a high discretization (e.g. 10°/bin) can be disruptive in a proportional control since decreases the amount of available information about stimulus (one can only distinguish at a more coarse scale). This loss of information is not incorporated into the measure that here we proposed. Evidently for such applications there should be a balance between stimulus discretization and amount of information calculated.

This evaluation can be carried out to determine the most adequate technique to be employed in a dynamical assistance myoelectrically-controlled device. Generally these devices use a set of parameters to form a feature vector, which is classified by using neural networks, artificial intelligence techniques, statistical methods and others [1]. However, the addition of parameters that do not provide information can lower the system performance. With the evaluation methods proposed in this paper is possible to evaluate and choose a set of parameters which provide a desired level of information.

All results here presented were obtained from the EMG recordings of all subjects. However, the choice of processing parameters (window length, processing technique, etc) for the implementation of a myoelectric control would have to be evaluated for each subject, because these would be applicable to only one user of prosthesis/orthotic and/or another assistive device. This implies that the correct choice of processing parameters should be performed according to variations over time (within to subject) and not to variations among subjects.

#### Abduction and adduction movements

The abduction and adduction movements are fundamentally different from a neuromuscular perspective one involves concentric muscle contractions the other eccentric contractions - eccentric contractions have been shown to have different motor unit recruitment [22]. However, this particularity could be expected in dynamic situations (moving arm) because to well-known abduction-adduction movement in which the deltoid muscle works as agonist during lifting of the arm losing protagonism when lowering it (of course, this means that other muscles increase their protagonism). As a consequence, the muscular effort is greater when the arm goes up than when it moves downward. Thus, changes in EMG amplitude would be accounted for by the latter at the same angular position when going up as compared to the opposite returning movement. In previous investigations, a quantifiable and significant middle deltoid EMG difference has been clearly demonstrated when the muscle is either ready to abduction or ready to adduction [13]. This effect was fully reproducible between 0 and 90° of an arm in static position within the scapular plane.

For static contractions the amount of information was (1.51 ± 0.33) [bit] when it was calculated in positions of 0° to 90° (positioning the arm with abduction movements) (Figure 7A). In a similar way, when the information is obtained from the positions of 90° to 0° (positioning the arm with adduction movements) was (1.55 ± 0.28) [bit]. However when the information is calculated using the arm positioning with two movements, this value decreased to (1.43 ± 0.32) [bit]. This decrease of the information average value is related to an increase of the variability of RMS values (Figure 7B). Our results show that this decrease was not significantly different. In other words, the arm positioning with abduction and adduction movements would not produce significant changes in the EMG signal of middle deltoid. The loss of protagonism of the middle deltoid when arm is lowering would not be significantly different from when is rising.

In dynamic contractions may also be observed a hysteresis cycle. The EMG amplitudes during abduction movements are greater than those obtained during adduction movements as in static contractions. The correlation between the arm position and EMG amplitude is higher during the abduction (higher information values) (Figure 7D). Contrary to what happened in static contractions, there is a significant change in the information (lower value) when the adduction movement is considered in independent form to the abduction movement. These results (both static and dynamic) should be expected given that response of the muscular system analyzed has a dependency with movement made (hysteresis). Therefore there would be a high probability that one of responses has a better correlation with the stimulus.

When both movements are considered in the information calculation is not observed a statistically significant variation, although the average value of information increases (Figure 7C). This increase of information could be contradictory since it would be expected that by including both movements the variability of the response increases, and therefore the information decreases (it which occurs if Abd is compared with Abd-Add). In this case, the increase of information (with respect to the movement of adduction) is due to that the variability of response introduced by abduction movement favors to correlation between the stimulus and response.

By using the evaluation methods proposed here would be possible to quantify the hysteresis effect given in muscular systems and their dependence on different experimental situations such as movements under external loads, speed of movement and muscle fatigue. Moreover, it would also determine the degree of correlation between the stimulus and response in different phases of movement. This latter study would be of particular interest in applications such as myoelectric control.

#### Inter-electrodes distance

In the past some researchers have attempted to determine the effect of some peripheral factors on EMG recordings. Merletti et al, (1999) investigated, using simulation techniques, the effect on inter-electrode distance on average rectified value and determined that the amplitude of the signal decays rapidly with increasing depth of the active muscle fibers for smaller values of inter-electrode distance [23]. By using a model for motor units, Fugelvand et al, (1992) simulated EMG and determined that inter-electrode distance can modify the amplitude of EMG [24]. Beck et al, (2005) analyzed the effects of inter-electrode distance on the absolute and normalized EMG amplitude versus isokinetic and isometric torque relationships for the biceps brachii muscle [25]. They found that inter-electrode distances between 20 and 60 mm resulted in similar patterns for the EMG amplitude versus dynamic and isometric torque relationships, and that the normalized EMG data were not influenced by changes at these inter-electrodes distances.

We observed an increase in amount of information with IED during abduction movements. This increase occurred for IED of 4 cm (Figure 7A and Figure 8) and it is due to a decrease in the variability of response (RMS values). The information values for IED of 6 cm did not change significantly with respect to the IED of 4 cm. In all cases, the information calculated from EMG recorded during adduction movements did not vary significantly with IED. These results reveal that the variation of IED significantly affects to abduction movements, while the correlation between stimulus and response is not affected during adduction movements. A behavior similar to abduction movement was observed when both movements were included in the calculation of the information. This result would be expected because the contribution of the response during the adduction movement on the amount of information stays invariant.

The variation of IED significantly affected to hysteresis cycle of middle deltoid (hysteresis cycle is less appreciable when inter-electrode distance increases). This effect is most noticeable when IED changes of 2 to 4 cm. In this situation there is an increase of information (Abd-Add) and is due to a decrease in the variability of response when IED increases.

Probably, very high IEDs are not beneficial for all muscles, since the EMG signals may be affected by crosstalk interferences. As mentioned above, the medium deltoid muscle is the main protagonist of abduction/adduction movements in the scapular plane and therefore was not affected by interference from other muscles. However, a flexion/extension arm which involves to the biceps muscle could be affected by interferences crosstalk.

The speed of abduction/adduction movements would have a significant effect on optimal segmentation length. Here, this phenomenon was not quantified because the aim of this paper is the proposal of the evaluation method. Experiments such as different loads and speed of muscular contractions may be of particular interest in the electrophysiology of muscle contractions, rehabilitation and/or control of prosthetics/orthotics through EMG signals, and others areas where the evaluation methods here proposed could be used for the signal processing.

## Conclusions

In this paper we proposed two methods based on information theory for the evaluation of processing techniques. Both methods were applied to different estimates of EMG amplitude such as RMS, AMV, DAMV and VAR. The window length is an important parameter for computing any EMG processing technique. The proposed methods allowed to solve the problem of EMG optimal segmentation. In static contractions the optimal window length was 200 ms and dynamic contractions was of 300 ms. In this paper we have shown that the RMS is the most appropriate technique for the analysis of EMG signals from muscle middle deltoid during abduction and adduction movements. Using this processing technique we analyzed the EMG activity under different experimental conditions. We have demonstrated, in static contractions that the difference between positioning the arm with abduction movements and adduction movements, it is not significant. In dynamic contractions were found similar results.

Although the evaluation methods were applied to standard processing techniques, these can also be considered as alternatives tools for evaluation of new processing techniques in different areas of electrophysiology (e.g. electromyography, electroencephalography, electroneurophysiology, and others).

## Competing interests

The authors declare that they have no competing interests.

## Authors' contributions

FDF and JCP designed and executed experimental protocols for acquisition of EMG recordings, participated in the data interpretation and drafted the manuscript. FDF conceived, designed and implemented the algorithms. CJF assisted in digital processing and data interpretation. All authors read and approved the manuscript.
